# Good practices for a “Decade for Active and Healthy Ageing”: Little drops make the mighty ocean Little Things, Julia Carney

**DOI:** 10.37825/2239-9747.1016

**Published:** 2020-10-01

**Authors:** M Illario, G Tramontano, V De Luca, O Piazza

**Affiliations:** 1 Dipartimento di Sanità Pubblica, Università degli Studi di Napoli Federico II, Naples, Italy; 2 Unità Operativa Semplice Ricerca e Sviluppo, Azienda Ospedaliera Universitaria Federico II, Naples, Italy; 3 Dipartimento di Medicina, Chirurgia e Odontoiatria “Scuola Medica Salernitana”, Università di Salerno, Salerno, Italy

## I. EDITORIAL

Ageing is a success story of Public Health worldwide, but poses many challenges that hinder its transformation into an opportunity for sustainable development: infectious outbreaks and public health emergencies, chronic diseases, social inequalities, environmental issues and more. Science and technologies provide a bulk of opportunities to implement innovative approaches, but their sustainability relies on our capacity of collaboration. Sharing a vision is a key step to drive us together into the future. The World Health Organisation (WHO) developed a proposal for “The Decade of Healthy Ageing (2020–2030)”, in the effort of bringing together governments, civil society, international agencies, professionals, academia, the media, and the private sector for ten years of concerted, catalytic and collaborative action to improve the lives of older people, their families, and the communities in which they live [1]. The proposal calls for concerted global action on Healthy Ageing and leave no one behind. According to WHO, Healthy Ageing is “the process of developing and maintaining the functional ability that enables wellbeing in older age. Functional ability is about having the capabilities that enable all people to be and do what they have reason to value”.

Implementing the goals of the WHO Decade 2030 for AHA can be facilitated by innovative approaches to health service provision, but a life-course approach urges that our efforts develop along multiple dimensions: the present special issue zooms on the measures used to assess the effectiveness of pioneers who explored new opportunities to improve health outcomes.

The physical dimension is not the only element influencing perceived well-being. Emerging barriers and the gender gaps influence well-being and resilience, impacting the older adult quality of life [2].

Cognitive function plays a key role in perceived well-being, and poses many challenges to the implementation of effective and innovative interventions. Group Reminiscence Therapy (RT) can have a positive impact on cognition, depressive symptoms, and quality of life in nursing homes older adults [3].

There are evidences on how early interventions can reduce the risk of stroke in patients with asymptomatic carotid stenosis (ACS). Professional training is a key element to ensure that prevention interventions are actually uptaken in current practice: sharing experiences and knowledge at international level is an opportunity we must not miss [4].

Integration of care means person-centered integration of health and social care, longitudinally provided across primary and secondary healthcare including citizens’ individual, social, economic and human resources [5].

Caregivers are key enablers in the adoption of the innovative approaches to integrated health and social service provision. Assessment of the caregivers’ satisfaction is pivotal to improve integrated care assistance and ensure the best quality of life for both patients and their families [6].

Each intervention, independently on the dimension, highlights a learning need to be addressed, that can be supported by informal caregivers. In order to develop trainings, it is important to take in consideration the role of the facilitator (volunteer or self-employed), the level of skills, the needs of the end-users, the training content and methodologies together with the sustainability of the learning [7].

Overall, this special issue drives the readers through some part of the multifaceted spectrum of AHA, raising -we hope- their curiosity and stimulating their efforts in integrating their current practice with those changes –as little or big they may be in their reach- that ensure they provide their contribution to the Decade too.
